# A deep-learning approach for automatically detecting gait-events based on foot-marker kinematics in children with cerebral palsy—Which markers work best for which gait patterns?

**DOI:** 10.1371/journal.pone.0275878

**Published:** 2022-10-13

**Authors:** Yong Kuk Kim, Rosa M. S. Visscher, Elke Viehweger, Navrag B. Singh, William R. Taylor, Florian Vogl

**Affiliations:** 1 Laboratory for Movement Biomechanics, Institute for Biomechanics, ETH Zürich, Zürich, Switzerland; 2 Laboratory for Movement Analysis, Department of Orthopedics, University Children’s Hospital Basel, Basel, Switzerland; Ningbo University, CHINA

## Abstract

Neuromotor pathologies often cause motor deficits and deviations from typical locomotion, reducing the quality of life. Clinical gait analysis is used to effectively classify these motor deficits to gain deeper insights into resulting walking behaviours. To allow the ensemble averaging of spatio-temporal metrics across individuals during walking, gait events, such as initial contact (IC) or toe-off (TO), are extracted through either manual annotation based on video data, or through force thresholds using force plates. This study developed a deep-learning long short-term memory (LSTM) approach to detect IC and TO automatically based on foot-marker kinematics of 363 cerebral palsy subjects (age: 11.8 ± 3.2). These foot-marker kinematics, including 3D positions and velocities of the markers located on the hallux (HLX), calcaneus (HEE), distal second metatarsal (TOE), and proximal fifth metatarsal (PMT5), were extracted retrospectively from standard barefoot gait analysis sessions. Different input combinations of these four foot-markers were evaluated across three gait subgroups (IC with the heel, midfoot, or forefoot). For the overall group, our approach detected 89.7% of ICs within 16ms of the true event with a 18.5% false alarm rate. For TOs, only 71.6% of events were detected with a 33.8% false alarm rate. While the TOE|HEE marker combination performed well across all subgroups for IC detection, optimal performance for TO detection required different input markers per subgroup with performance differences of 5-10%. Thus, deep-learning LSTM based detection of IC events using the TOE|HEE markers offers an automated alternative to avoid operator-dependent and laborious manual annotation, as well as the limited step coverage and inability to measure assisted walking for force plate-based detection of IC events.

## Introduction

Clinical gait analysis is a key tool for monitoring neuromotor deficits in pathologies such as Cerebral Palsy (CP) or Parkinson’s disease. By quantitatively providing kinematic and spatio-temporal measures, gait analysis supports clinicians in diagnosis and treatment decisions [1, 2]. Clinical gait analysis allows the characterisation of specific movement and functional deficits and has become an established approach in paediatric medicine for assessing movement disorders. To quantify these deficits across individuals, ensemble-averaged kinematics signals over the course of a gait cycle are used [1–6]. This process is critically dependent upon the accurate identification of a gait cycle. A gait cycle is most often defined using gait events such as initial contact (IC) and toe-off (TO), which discriminate between gait phases (stance vs swing). Incorrect identification of gait events could lead to errors in ensemble averaging of joint angles and inaccurate spatio-temporal parameters.

In current clinical practice, clinics are equipped with either an optical motion capture system (OMCS), force plates, or both. IC and TO are most often detected when the vertical component of the ground reaction force (vGRF), measured by force plates in an instrumented walk way [4–6], exceeds a certain threshold. Clinics and research institutes use different vGRF thresholds due to a lack of standardisation, however, this difference in vGRF threshold does not lead to meaningful differences [4]. Unfortunately, vGRF thresholding does not always work well; for example, it fails to recognise the next consecutive step if a foot is already placed on a force plate, thus demanding one step at a time. Achieving clean force plate hits is not always possible in the case of paediatric subjects and/or pathological gait patterns; due to short leg-length they may simultaneously have multiple steps on the same force plate, requiring manual annotation from trained experts, which is time-consuming and operator-dependent. Furthermore, the force threshold technique is not applicable for assisted gait, where participants with severe movement disorders might walk with crouches or a walker. In addition, the costs of force plate installation and maintenance are expensive and limit the number of steps available for analysis per measurement.

For these reasons, kinematic approaches to detect gait events have been introduced as an alternative to force-plates or manual annotation. These kinematic approaches detect the timing of gait events by considering the position, velocity, or acceleration of body segments, which can all be measured using e.g. inertial measurement units (IMUs) or OMCS [7–27]. Majority of IMU studies for gait event detection are based on either gyroscope or accelerometer [13–17, 23–27], despite the portability and accessibility, due to the characteristic of IMUs, extracting as accurate kinematic data as to OMCS are challenging thereby limiting the application of IMUs for standard clinical gait assessment. OMCS is therefore still the most applied methods for collecting kinematics during clinical gait assessment to assist clinical decision making. So far, most works on kinematic detection algorithms based on OMCS have focused on cohorts with physiologically normal gait patterns, in which a heel strike constitutes the IC [7–12]. However, many pathologies are associated with modified walking patterns, such as crouch-gait in CP or toe-walking in idiopathic toe-walkers [28, 29]. While some studies have investigated developing kinematic algorithms based on OMCS suited to specific gait patterns with pathological groups [18–22], and demonstrated promising results, there is only a handful of studies comparing how the tailored kinematic algorithms perform on other gait patterns [4, 10, 12]. In clinical practice, wide various gait patterns are assessed daily. Therefore, it is practical to evaluate how these methods are generalisable across multiple gait patterns.

Based on the results of the 2019 SOFAMEA challenge, which was a contest to design the best automatic approach to detect gait events based on kinematic inputs, deep-learning long short-term memory (LSTM) appears to be the most promising method for assessing pathological gait patterns [30, 31]. Even though the SOFAMEA dataset consisted of kinematic data collected from CP subjects with different gait patterns (heel strike, crouch, toe-walking), no categorisations were provided. Consequently, it was impossible to evaluate the performance on these individual gait groups. Furthermore, considerable differences exist in the marker numbers and locations for measuring the kinematics across movement laboratories. Therefore, it is of particular practical relevance to evaluate how the performance of a detection algorithm changes when using different marker inputs, and whether certain inputs perform better than others for specific gait patterns. This study measured and categorised 363 participants with CP based on the part of the foot that was involved in IC (Forefoot, Midfoot, or Heelstrike). We then developed a deep-learning approach based on foot-marker kinematics to detect IC and TO and a) compared its performance across these three gait patterns, and b) studied what combination of marker inputs optimised the performance for each subgroup.

## Methods

### Data

**Fig 1:**
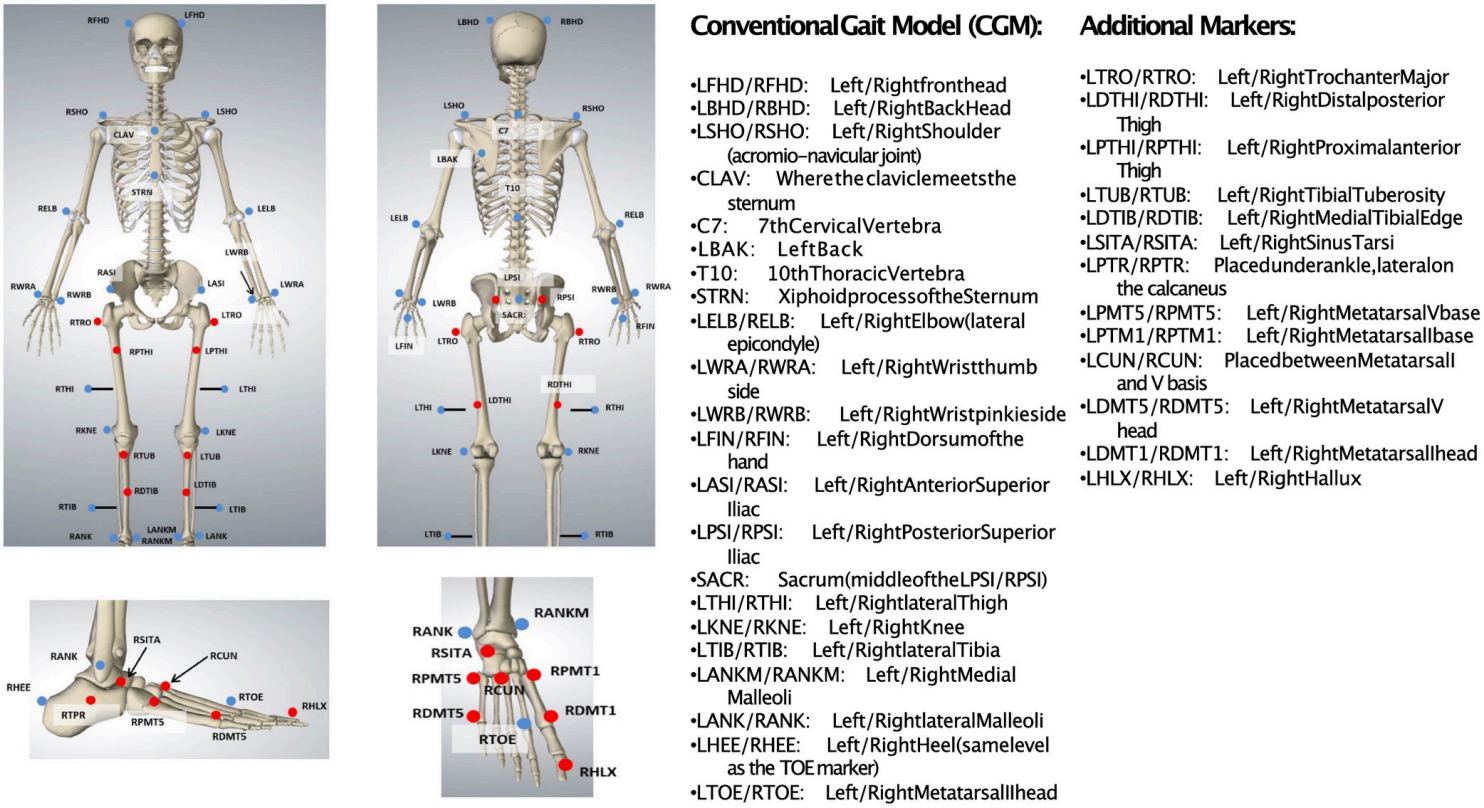
An overview of modified Conventional Gait Model.

This study used a retrospective database consisting of 3D kinematic and kinetic data of 363 participants with movement disorders classified as Gross Motor Function Classification System (GMFCS) levels I to III [32], aged 3-18 (11.8 ± 3.2) years at the time of measurement, who underwent 3D gait analysis at one hospital between 2015 and 2017. All these participants could complete a barefoot walking trial without orthopaedic devices, crutches or walkers. Informed consent was obtained from all children or their guardians, under approval from the local ethical committee (KEK BASEC-Nr. 2018-01640). All measurements were conducted according to the Declaration of Helsinki. This investigation started in September 2019 and was completed in November 2021. Collected data was pseudo-anonymized, table to connect research ID with patient ID was stored securely on the hospital server. The 3D kinematics were collected using a twelve-camera optical marker system (MTX20, VICON, Oxford, UK) at a sampling frequency of either 150 Hz (after 2016) or 300 Hz (before 2016). Kinetic data were collected at a sampling frequency of 1500 Hz using four force plates (Kistler, Winterthur, Switzerland) embedded in the middle of the walkway. All participants were fitted with a modified Conventional Gait Model (CGM) [33], with 64 markers of 9.5mm diameter each showing in Fig 1.

**Fig 2:**
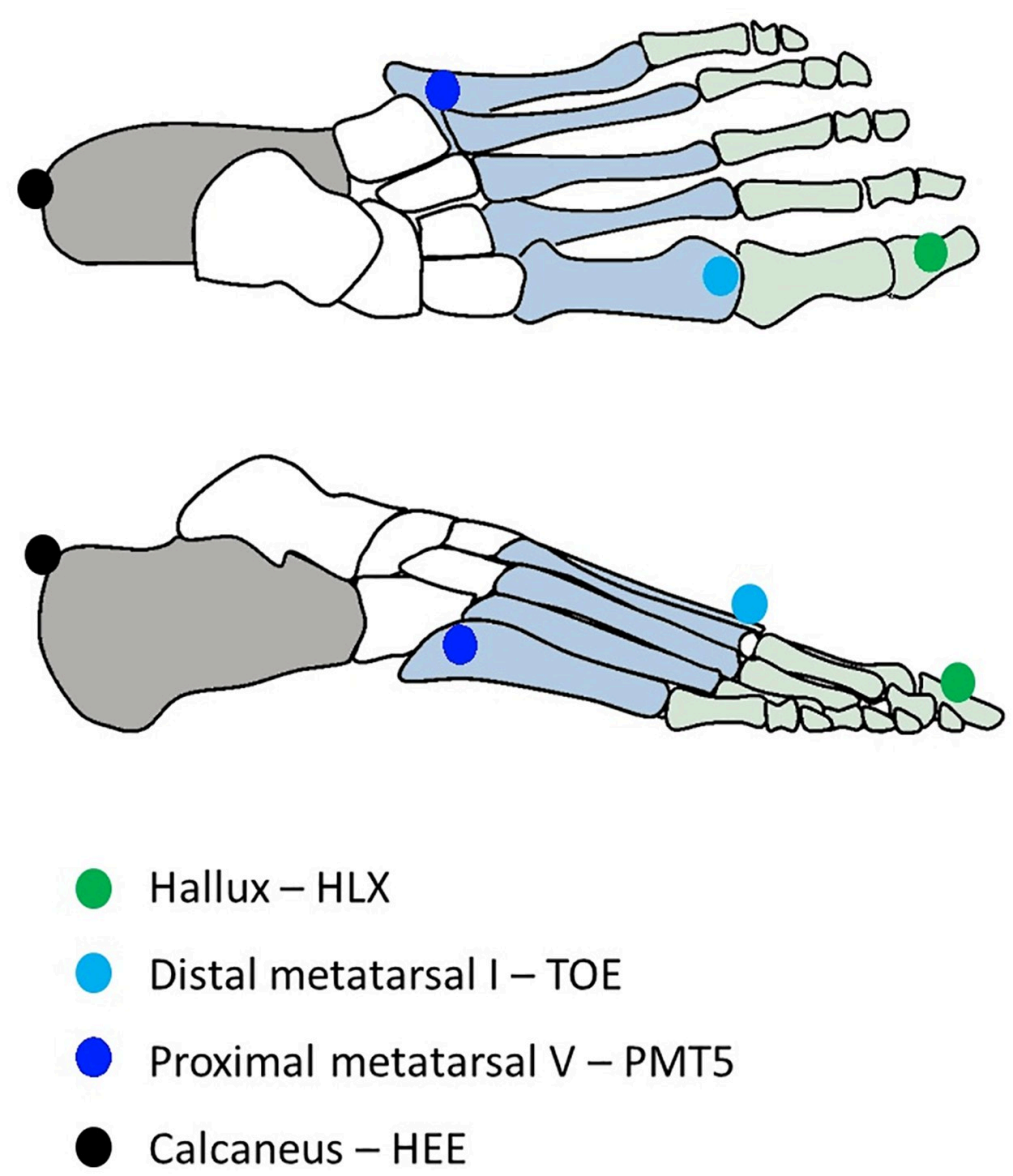
An overview of foot markers used within analysis. The grey area indicates the part of the foot used by the HS group for IC, the blue area the part of the foot used by the MF group for IC, and the green area the part of the foot used by the FF group for IC. HS: heel-strike, IC: initial contact with the ground, MF: midfoot, FF: forefoot.

Whenever marker data from the calcaneus (HEE), distal metatarsal I (TOE), hallux (HLX), or proximal metatarsal 5 (PMT5), markers were missing due to occlusions, these trials were excluded. Please refer to Fig 2 for the foot markers.

The marker positions *s*_*n*_ were downsampled to 150 Hz, and the marker velocities *v*_*n*_ were calculated by

$$\begin{array}{c} v_{n}=\frac{s_{n+1}-s_{n}}{\Delta t}, \end{array}$$ (1)

where *n* denotes the frame and Δ*t* = 1/150 s.

All participants were classified into three groups according to the foot region that initially contacted the ground:

1. A forefoot (FF) group: consisting of toe-walkers with forefoot contact.
2. A midfoot (MF) group: flat-foot walkers for whom the entire sole or the side of the foot touched the ground.
3. A heel (HS) group: typically developed gait patterns with initial heel-strike contact.

**Fig 3:**
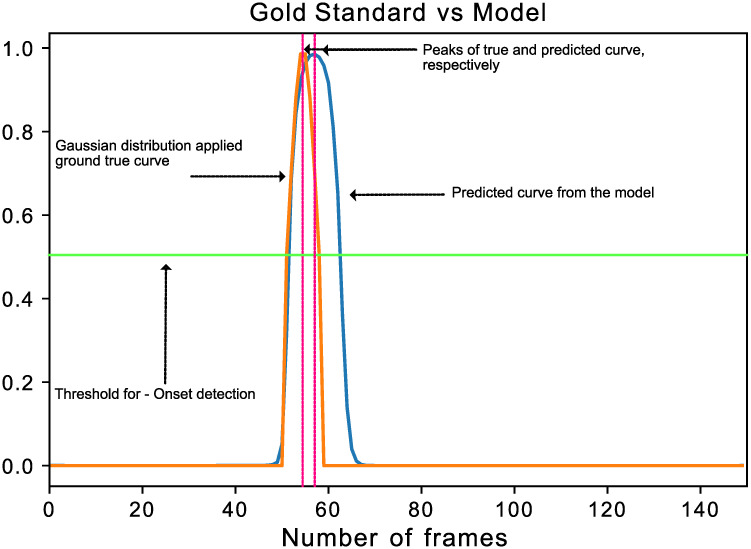
One advantage of our algorithm is that it is not binary in nature. Instead of just giving a peak event location, it outputs a curve. So it might be advantageous to consider an event as a time duration (e.g. the Full-width-at-half-maximum of the peak) instead of just timepoint (e.g. found through peak detection in this work).

The time-points of foot contact events were found by setting a threshold of 20N [4] for the vertical ground reaction force or by visual identification from video data by an experienced operator when force plate data was unavailable. Here, it is noteworthy that manual annotation suffers from inter-rater variability, which one study found to be less than 16ms in 84% of events compared to a gold standard rater [3]. To account for the uncertainty in the true gait events times, we modelled gait events as a Gaussian probability distribution at the nominal event times with a standard deviation of 16ms as shown in Fig 3. This procedure yielded 5340 IC and 4937 TO events with a 4:2:1 ratio between heel, midfoot, and forefoot groups, considered the ground truth.

The dataset was split into a training-(80%), a validation-(10%), and a testset-(10%), with statistics shown in Table 1. Notably, each subject was only part of one of these three subsets to avoid the network over-fitting to the subject-specific walking patterns. Furthermore, the testset was identical to a previous study that investigated kinematic algorithms to allow for direct comparison [1]. Lastly, ten training runs were performed with different training/validation dataset splits and evaluated on the same testset.

**Table 1:** Participant description.

	FF-Group	MF-Group	HS-Group
**Part(s) of the foot in contact with the ground during IC**	The forefoot	The entire sole/the side of the foot	The heel
**Amount of trials used to create Training- and Validation-set**	N = 96	N = 217	N = 413
**Sagittal joint angles at IC in degrees**			
**Ankle-dorsiflexion angle [Mean (SD)]**	-16.5 (12.1)	-5.4 (7.3)	-1.9 (4.9)
**Knee-flexion angle [Mean (SD)]**	14.7 (12.4)	9.6 (10.6)	5.0 (5.6)
**Hip-flexion angle [Mean (SD)]**	37.1 (12.2)	34.2 (10.2)	34.4 (8.2)
**Age in years [Mean (SD, range)]**	12.4 (3.1, 6.6-17.6)	12.5 (3.2, 6.3-17.9)	12.6 (3.2, 5.6-17.7)
**Sex [M/F]**	53% / 47%	59% / 41%	59% / 41%
**GMFCS level [I / II / III]**	62% / 32% / 6%	84% / 16% / 0%	90% / 10% / 0%
**Diagnosis**			
**Neurological [CP-uni / CP-bi / Other]**	24% / 28% / 21%	20% / 17% / 22%	11% / 6% / 12%
**Skeletal [Malalignment / deformity]**	6% / 9%	8% / 24%	14% / 45%
**Muscle [Myopathy]**	8%	3%	4%
**Other [Genetic / ITW]**	4% / 0	15% / 1%	38%/ 0%
**Test-set**	N = 30	N = 30	N = 30
**Sagittal joint angles at IC in degrees**			
**Ankle-dorsiflexion angle [Mean (SD)]**	-17.0 (11.8)	-2.6 (7.8)	-1.1 (5.7)
**Knee-flexion angle [Mean (SD)]**	17.9 (13.8)	16.6 (7.6)	11.6 (10.4)
**Hip-flexion angle [Mean (SD)]**	32.5 (13.7)	30.9 (10.6)	32.0 (9.0)
**Age in years [Mean (SD, range)]**	11.2 (3.2, 5.9-17.3)	11.6 (3.4, 5.6-17.3)	12.6 (2.6, 7.7-17.6)
**Sex [M/F]**	50% / 50%	60% / 40%	60% / 40%
**GMFCS level [I / II / III]**	57% / 40% / 3%	83% / 17% / 0%	97% / 3% / 0%
**Diagnosis**			
**Neurological [CP-uni / CP-bi / Other]**	23% / 33% / 13%	43% / 13% / 10%	7% / 17% / 0%
**Skeletal [Malalignment syndrome / foot deformity]**	7% / 7%	14% / 7%	43% / 27%
**Muscle [Myopathy]**	7%	3%	3%
**Other [Genetic / ITW]**	3% / 7%	10% / 0	3% / 0

IC: initial contact; SD: standard deviation; M: male, F: female, GMFCS: gross motor function classification system the higher the level the more severely impaired the motor function is in the affected individual, CP: cerebral palsy; uni: unilateral, bi: bilateral, Neurological-Other: traumatic brain injury, incomplete spinal cord injury, infection, tumour; ITW: idiopathic toe-walking.

### Network architecture, training, and evaluation

Long short-term memory (LSTM) is an advanced network of recurrent neural networks (RNN), which may be able to utilise past information to help carry out the task in the present [34]. However, the length of the past using RNN is limited, whereas LSTM can store the information for a more extended time. This study employed LSTM for three main reasons, 1) the inputs are 3D time-varying kinematic data, 2) LSTM is entrenched in the research 3) two previous studies were based on using LSTM. Hence, to compare the result, LSTM was chosen.

The network consisted of a bidirectional LSTM, followed by a fully connected layer and a sigmoid activation function. The bidirectional variant of the LSTM allowed the network to consider the information before and after the event to make its predictions—with the drawback that the network can not be used for real-time detection. To counteract the imbalance in the dataset, we sampled the gait groups in a 4:2:1 ratio. Each sample consisted of the 3D marker positions and velocities from exactly one gait event, cut to a length of 150 frames around the true event with a random offset within the range of ±30. The model was trained with an Adaptive Moment Estimation (ADAM) optimiser and a binary-weighted cross-entropy loss, while the learning rate, the number of neurons in the fully connected layer, the number of LSTM layers, and the drop-out rate were included as hyper-parameters Table 2). Models underwent the same set of hyper-parameters to compare the impact of input features objectively. In other words, each model was not optimally tuned to outperform others, but the model was selected for the highest performance with the given hyper-parameters set. The network was implemented using the PyTorch library on a Windows 10 (64-bit) computer. The model was trained and tested on a computer with a 3.6GHz CPU AMD Ryzen 7 3700X 8-Core Processor, 32GB RAM, and an Nvidia GTX 2080 GPU card. All codes and further details regarding hyper-parameters can be found on our GitHub (https://github.com/ykukkim/W04_DL).

**Table 2:** Hyper-parameter set for optimum performance.

Input Markers—IC	No.of.input features	No.of LSTM layers	No.of Hidden size	Drop out	Learning rate
HLX|HEE	12	2	512	0.3	0.001
TOE|HEE	12	5	256	0.3	0.001
HLX|PMT5|HEE	18	2	256	0.3	0.001
TOE|PMT5|HEE	18	5	256	0.3	0.001
HLX|TOE|HEE	18	2	512	0.3	0.001
Input Markers—TO	No.of.input features	No.of LSTM layers	No.of Hidden size	Drop out	Learning rate
HLX|HEE	12	5	256	0.3	0.001
TOE|HEE	12	2	512	0.3	0.001
HLX|PMT5|HEE	18	2	256	0.3	0.001
TOE|PMT5|HEE	18	5	256	0.3	0.001
HLX|TOE|HEE	18	2	256	0.3	0.001

During the evaluation, a standard peak detector (scipy.signal.find_peaks) predicted the time-point of gait events from the network output, which was a time series of values between 0 and 1. Only peaks higher than 0.5 were considered gait events, and for each predicted gait event, we calculated the time difference to the corresponding true event. Whenever a predicted peak fell within ±16ms of the true event, this was considered a successful prediction, while predictions further away were deemed to be false alarms. Similarly, we calculated how many true events had at least one predicted gait event within 16ms, and how many true events were missed.

## Results

Our deep learning model predicted between 78.5% and 93.9% of true IC events (between 58.5% and 81.4% for true TO events) depending on gait group and input marker combination (Table 3). Between 6.1% and 21.5% of true IC events were missed (between 18.6% and 41.5% of TO events). Similarly, between 71.1-89.7% (IC) and 54.4-76.9% (TO) of predictions represented real events, and between 28.9%-10.3% (IC) or 45.6-23.1% of predictions were false alarms (Table 4)—again with substantial variations depending on gait group and marker inputs.

**Table 3:** Percentage of true events that have a corresponding *predicted* event within ±16ms.

	Initial Contact				Toe-Off			
Input Markers	Forefoot	MidFoot	Heel	Overall	Forefoot	MidFoot	Heel	Overall
HLX|HEE	80.3±3	78.5±2.7	92.8±1.8	83.8±1.8	64.3±2.2	**67.7±2.8**	78.2±1.7	70.1±1.7
TOE|HEE	**90.8±2.1**	**85.4±2**	93.2±2.2	**89.7±1.3**	**76.4±3.5**	58.5±3.5	75.5±3.1	70.1±2.4
HLX|PMT5|HEE	88±2.4	81.8±2.2	**93.9±0.9**	87.9±1.4	71±1.7	62.3±2.8	**81.4±1.3**	**71.6±1.8**
TOE|PMT5|HEE	88±4.1	84.8±1.7	93.2±1.6	88.7±1.7	69.8±2.2	62.8±3.7	73.1±2.5	68.6±1.8
HLX|TOE|HEE	82.3±2.4	83±2	92.5±1.6	85.9±1.4	74.5±1.8	58.6±1.9	79.5±2.5	70.9±2

Presented are the mean and standard error of the mean, evaluated over ten models (trained on different training/validation splits) on the same testset. Best performances within a gait group are printed in bold font.

**Table 4:** Percentage of predicted events that correspond to a *true event* within ±16ms.

	Initial Contact				Toe-Off			
Input Markers	Forefoot	MidFoot	Heel	Overall	Forefoot	MidFoot	Heel	Overall
HLX|HEE	71.1±2.4	70.4±1.5	88.5±1.5	76.9±1.9	58±1.8	**60.6±2.3**	72.9±1.6	63.8±1.6
TOE|HEE	**80.4±2.9**	**79.5±1.4**	88.7±2.2	**82.5±1.5**	**66.9±1.9**	58.8±3.3	71±2	65.6±1.7
HLX|PMT5|HEE	76±2.4	76.6±1.1	**89.2±1.6**	80.6±1.5	65.7±1.5	56.1±2.4	**76.9±1**	**66.2±1.9**
TOE|PMT5|HEE	76±3.1	77.9±1.2	87.7±1.1	80.5±1.5	64.7±1.9	54.4±2.7	69.8±1.9	63±1.7
HLX|TOE|HEE	74.6±2.3	76.1±2	**89.7±1.3**	80.2±1.6	**67.9±1.7**	55.5±2	73.8±1.3	65.7±1.7

Presented are the mean and standard error of the mean, evaluated over ten models (trained on different training/validation splits) on the same testset. Best performances within a gait group are printed in bold font.

**Fig 4:**
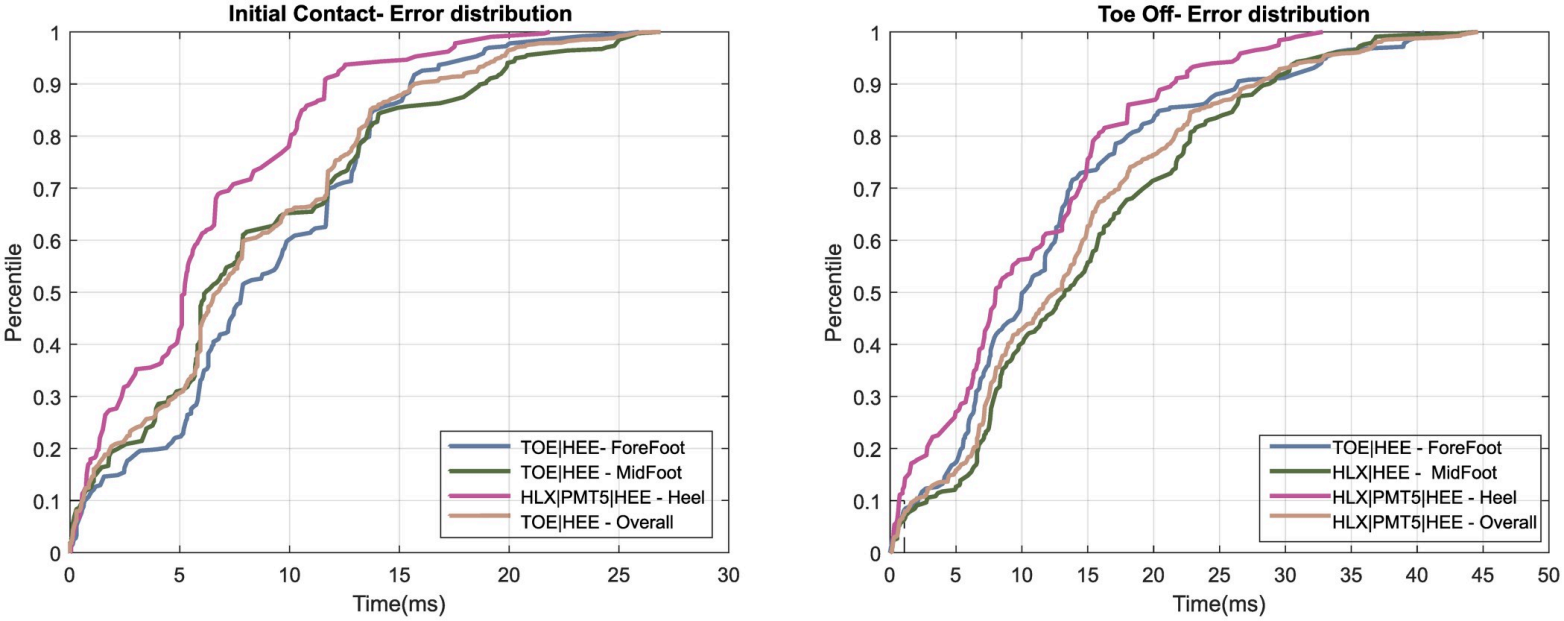
Percentile plot of the absolute prediction error in milliseconds of the best performing model for each gait group over the testset.

Fig 4 displays the absolute prediction error over the testset for each gait group’s best performing marker combination. IC detection was more successful for the HS group than for MF or FF group, with MF predictions being better than FF predictions for about 80% of the samples. For TO detection, our algorithm once again performed best for the HS group, but this time performance was better on FF walkers than on MF walkers.

Shown are the mean performances of ten models (for each subgroup and corresponding marker combination) trained on different training/validation splits and evaluated on the same testset. Irrespective of input marker combination, the training variability estimated over 10 training runs with different training/validation dataset-splits (Tables 3 and 4), which were in the order of 2%- 4% for all input markers and all gait groups.

The prediction error rose linearly over a large percentage of the test-set before reaching a plateau, indicating that only a few samples were badly mispredicted. Notably, the fraction of predictions within 16ms of the true value increased quickly with allowable error (Table 3). For example, the TOE|HEE model predicted 85% of IC events to within 15ms, its detection rate increased to about 95% for events within 20ms (Fig 4).

## Discussion

In this study, we compared the performance of a deep learning algorithm across various walking patterns and different marker combinations. We specifically focused on the practical questions of a) how does our deep learning approach compare to manual annotation, automatic event detection using kinematic algorithms, and automatic detection using other deep learning approaches, and b) how well does our approach perform for different gait groups and is there an advantage of using specific input markers for a particular subgroup? We considered a performance difference of 3 percentage points as the relevancy limit for practical applications for the rest of this discussion.

### How does the performance compare to the manual annotation of gait events, automatic prediction using kinematic algorithms, and other deep learning approaches?

Our approach correctly predicted 89.7% of true IC events for the overall gait group (Table 3), and 82.5% of all predicted IC events corresponding to real events (Table 4). Overall, these results mean that about 10% of real IC events would be missed in practice, and 18% of predictions would be false alarms. TO detection proved to be more difficult than IC detection, with only about 71.6% of true events detected successfully for the overall group and 66.2% of TO predictions corresponding to real events. This difference is unsurprising as manual annotation of TO is known to be challenging compared to IC [30, 31]. Notably, only a small fraction of events was predicted with high errors (Fig 4); thus, an additional 5ms error (i.e. a total of 21ms) increased the fraction of successfully detected events from 85% to about 93% for IC, and from 75% to 85% for TO. Such an additional error margin of 5ms would be negligible for most applications. In the face of these high detection rates, automatic detection appears preferable to the laborious and operator-dependent manual annotation for most practical applications (see below for alternative automatic approaches).

Our approach’s performance was comparable to the kinematic algorithms developed for event detection in pathological gait. On the same test-set, the best performing kinematic algorithm was capable of identifying 95% of IC and events within 33ms and 22ms [4], whereas our approach these events to within 20ms and 33ms, respectively (Fig 4). When optimised towards specific gait patterns, 95% of the events were detected within 7ms and 12ms for IC and TO respectively [22]. While our approach showed varying performance per subgroup, the kinematic algorithms only showed performance differences when optimised on a particular subgroup. Kinematic algorithms might therefore be preferable for measuring cohorts with homogeneous pathological gait patterns. It might also be possible to train the ML model on each specific sub-group rather than across all groups (specialised ML). Future research would be needed to investigate whether such a specialised ML model could outperform an optimised kinematic algorithm. As kinematic algorithms require optimisation for each gait pattern and are dependent on manually chosen thresholds, which only makes them semi-automatic, ML approaches could be preferable for labs that measure various gait patterns.

Two previous studies investigated automatic event detection based on machine learning using similar methods to our approach and reached similar detection performances. One study trained a LSTM on a dataset of 18153 trials (the number of IC and TO events are unknown) and reported a 99% trimmed-average time difference of 18.3ms for IC detection, and a 95% trimmed-average time difference of 12.5ms for TO detection [30]. Another study trained a bi-directional LSTM on 1156 trials (10526 IC, 9375 TO) and reached average time errors of 5.5ms and 10.7ms for IC and TO, respectively [31]. Our method was trained on 1732 trials (5340 IC, 4937 TO) and resulted in an 95% trimmed-average time error of 8ms (IC) and 11.2ms (TO). The similar performance between these two studies and ours, indicates that deep learning methods for event detection can be applied successfully using comparably smaller datasets such as ours, thus opening perspectives for smaller laboratories to develop custom approaches. Unfortunately, the datasets of these two studies were not sub-divided into gait groups, making a more detailed comparison impossible—future studies should investigate whether these larger datasets would be beneficial for detecting gait events for FF and MF groups, where our approach showed a reduced detection performance.

Our results regarding different marker input combinations exhibited a minor role (in the order of 5%) for the performance on the overall group. (Tables 3 and 4). While the previously mentioned two studies and ours used slightly different model inputs (addition of hip and knee joint angles, other choice of marker-sets), the comparable performance of all approaches indicates that all of these inputs provide the same core information. Surprisingly, the bi-LSTM study stated that the length of input data is an important input parameter, and claimed that the implementation of a zero-padding technique improved the performance [31]. Why the authors claim that this zero-padding technique, which does not add any information, would improve performance remained undiscussed. Thus, it seems that the slight technical differences (sampling frequency, length of the input, time-series, hyper-parameters) between the three studies do not have a major impact on the performance. Consequently, these methods of gait event detection seem robust and translatable to other labs that have slightly different settings.

Practically, it is important to not only consider the fraction of true events that are detected by the algorithm, but also how many of the detected events corresponded to real events—and how many events were false alarms/ghost events. Especially when using detected events as experimental triggers (e.g. for electrical stimulation during walking), such false alarms must be kept to a minimum. For our approach, about 10% of detected IC-events were false alarms for the HS group, and about 20% for MF and FF groups (Table 4). Once again, TO events were harder to detect, leading to higher false alarm rates of about 25% for the HS group, 40% for the MF group and 35% for the FF group. To our knowledge, this study is the first to consider the false detection rate, making it difficult to compare this metric with other work.

### How well does the algorithm perform on different gait groups and is there an advantage to using certain input markers when using deep-learning based predictions?

This study evaluated the effect of using different markers as inputs for deep learning based models and evaluated the performance on three different gait groups. Our approach detected about 90% of IC events for the overall group, with the best performance in the HS group (93.9%), followed by the FF group (90.8%), and the MF group (85.4%). As discussed above, detection performance was worse for TO than for IC, with our algorithm detecting 71.6% of TO events in the overall group, 81.4% in the HS group, 75.4% in the FF group, and 67.7% in the MF group. The fact that IC detection showed a higher performance for the HS group than for FF or MF groups is unsurprising, given that this group had the highest amount of data and because this group displays a walking pattern that is most similar to healthy gait with a heel strike, which is known to have low variability between subjects.

The high variability in heel-toe progression known to be present in MF, could be the reason for the MF group showing the worst performance for both IC and TO detection. Additionally, our dataset only classified MF indirectly—while, HS and FF were clearly defined based on their dorsi-flexion angles during IC (section Methods), all cases which did not correspond to the criteria for HS or FF were classified as MF [22]. The fact that we chose subgroups based on IC could also be one reason why the TO detection showed a higher inter-group variability than the IC detection. Thus, it might be beneficial to further investigate how to improve categorisation of gait groups and perhaps categorise TOs separately.

Depending on the input markers, the performance on the overall group differed by up to 5.9% for IC detection and up to 3% for TO detection (Table 3). When looking at individual gait groups, the IC detection rates varied by 10.5%(FF group), 6.9% (MF group), and 1.4%(HS group), while the TO detection rates varied by 8.3% (HS group), 12.2%, (MF group), and 12.1% (FF group).

While the addition of PMT5 improved detection performance in comparison to HLX|HEE for most cases, it was surprising that the addition of PMT5 to TOE|HEE generally decreased performance (MF group being exceptions in both cases). Similarly, the performance of HLX|TOE|HEE unexpectedly lay between those of TOE|HEE and HLX|HEE. These results clearly show that simply adding more information is not always beneficial and can sometimes even worsen performance.

Overall, our results suggest that different marker inputs perform similarly when detecting IC events in the HS group, but lead to considerable detection differences for other gait groups or for TO detection. However, the consistently high performance of TOE|HEE shows that this marker combination is an excellent choice for identifying IC gait events across gait patterns. For TO detection, no marker combination performed well over all groups—here it is advised to choose TOE|HEE for FF, HLX|HEE for MF, and HLX|PMT5|HEE for HS and the overall group.

### Limitations and outlook

Our cohort consisted of children with CP, so our results might not be generalisable to other pathologies, age groups or movement patterns. The unbalanced dataset might also have limited the ability of the algorithm to generalize to unseen data for the less well represented subgroups (FF and MF group). Our categorisation into subgroups is also imperfect, as the HS and FF group were clearly defined based on their dorsi-flexion angles during IC (section Methods), while everything else was classified as MF. The MF group might thus have a higher inhomogeneity than the other groups. The ground truth event annotations were partly subject to operator-variability for manually annotated events and to variability stemming from the detection threshold for events annotated by force measurement. The use of manual event identification was unavoidable in case of dragging of the feet and extremely short step length, which are often present in paediatric pathological gait. To avoid mislabelling, all manual annotations were performed by trained gait laboratory personnel and verified by a scientific assistant. This additionally manual annotation was required for a third of the dataset.

Judging by the differences in performance on the individual gait groups, it is likely that specialized networks would perform better than the generic network trained in this study. For kinematic algorithms, the selection of optimum input markers for each gait group improved the event detection from 80ms to 7ms for IC and from 143ms to 12ms for TO detection [22]. In clinical practice, such a differentiation of networks would mean that every subject would need to be categorised by their dominant walking pattern prior to gait analysis in order to choose the optimal marker set for detecting gait events.

As our study only investigated event detection on pathological gait barefooted, it remains unclear how well our approach would perform for detecting gait events in pathological gait with shoes and healthy subjects. An evaluation of our approach to healthy subjects would be needed to align data processing between pathological and healthy cohorts for research purposes. Considering other studies [12, 21] found that automatic event detection with kinematic algorithms was more successful for a healthy cohort than for a CP cohort (corresponding to our overall gait group), due to narrower gait variations such as walking speed in healthy cohort, we expect our approach might perform even better when used for detecting gait events in healthy subjects.

LSTM is a well-established model across various fields and has shown to be successful in delivering high performance. Still, in recent years, there have been new models that potentially reach better performance, such as applying the attention model in LSTM. Furthermore, several studies [35–40] have also been conducted on hybrid deep learning architecture in other fields. Thus, one could speculate that using such a new model could enhance performance, and might therefore be worth to evaluate in a future study.

## Conclusion

This study developed a deep-learning approach based on foot-marker kinematics to detect IC and TO in a CP cohort, and evaluated how its performance changed across three gait subgroups when using different marker inputs. For the overall group, our approach detected 89.7% of IC with a 18.5% false alarm rate, but only 71.6% of TO events with a 33.8% false alarm rate. The performance on the three different subgroups differed by about 5%-10%. For IC detection the TOE|HEE marker combination performed well across all subgroups, but optimal performance for TO detection required using different input markers (TOE|HEE for FF, HLX|HEE for MF, and HLX|PMT5|HEE for HS, with performance differences of 5-10%). Thus, automatic detection of IC using the TOE|HEE marker offers perspectives to avoid the labour and operator-variability associated with manual annotation, as well as mitigate possible limited step coverage and inability to measure assisted walking for force plate-based detection of IC events.
